# Progression of malignant pleural effusion during the early stage of gefitinib treatment in advanced *EGFR*-mutant lung adenocarcinoma involving complex driver gene mutations

**DOI:** 10.1038/s41392-020-0161-7

**Published:** 2020-05-27

**Authors:** Ning Liu, Min Yu, Tao Yin, Yong Jiang, Xuelian Liao, Jie Tang, Yanying Li, Diyuan Qin, Dan Li, Yongsheng Wang

**Affiliations:** 1 Department of Thoracic Oncology, State Key Laboratory of Biotherapy and Cancer Center, West China Hospital, Sichuan University and Collaborative Innovation Center, Chengdu, 610041 China; 2 Department of Pharmacology and Cancer Biology, Duke University, Durham, NC USA; 3 Department of Pathology, West China Hospital, Sichuan University, Chengdu, 610041 China; 4 Frontier Science Center for Disease Molecular Network, State Key Laboratory of Biotherapy, West China Hospital, Sichuan University, Chengdu, 610041 China; 5 Precision Medicine Center, Precision Medicine Key Laboratory of Sichuan Province, West China Hospital, Sichuan University, Chengdu, 610041 China; 6 Institute of Drug Clinical Trial, West China Hospital, Sichuan University, Chengdu, 610041 China

**Keywords:** Cancer genetics, Lung cancer

**Dear Editor,**

Malignant pleural effusion (MPE) has been reported in ~40% of advanced non-small cell lung cancer (NSCLC) patients, most frequently in those with lung adenocarcinoma (LUAD). Epidermal growth factor receptor tyrosine kinase inhibitors (EGFR-TKIs) have brought a significant improvement in the clinical treatment of advanced *EGFR*-mutant NSCLC, however, during the early stage of EGFR-TKI administration, progression of MPE rather than lung lesions has been observed in a small group of patients. Although defined as a nontarget lesion by the Response Evaluation Criteria in Solid Tumors (RECIST) guidelines (version 1.1), MPE represents true neoplastic pleural dissemination and worse prognosis. In this situation, some clinicians tend to give up targeted therapy and switch to chemotherapy, since it seems that the patients do not benefit from EGFR-TKI treatment. Therefore, it is necessary to explore the underlying mechanism of early MPE progression for better clinical decision-making.

This study was approved by the West China Hospital of Sichuan University Biomedical Research Ethics Committee, and informed consent was waived. From 2016 to 2019, five patients with *EGFR*-mutant LUAD accompanied by small amounts of MPE who developed pleural effusion progression within approximately 1 month after first-line gefitinib treatment (250 mg per day) were recruited (Supplementary Table S1). The response of lung lesions to gefitinib was assessed as stable or unevaluable because it was difficult to distinguish between the compressed lung tissue and tumor. MPE progression was confirmed by chest CT (Fig. 1a) and cytologic examination (Fig. 1b). Continuous gefitinib treatment and pleural effusion drainage followed by intrapleural cisplatin (DDP) administration (60 mg) were performed since there was only evidence of MPE progression. Patient 4 was not given DDP because of renal insufficiency. CT scans were conducted every 2 to 3 months until disease progression or death. The objective response (OR) of the MPE was evaluated according to a standard described previously.^1^ All five patients achieved MPE control, including 1 complete response (CR) and 4 partial responses (PRs) (Fig. 1a). Three patients remained stable at follow-up, and the other 2 patients had pleural effusion recurrence at 11 and 19 months after treatment (Supplementary Table S2). All patients benefited from continuous gefitinib treatment. The median progression-free survival (PFS) was 8.8 months (ranging from 7.8 to 19) (Fig. 1c), and the median overall survival (OS) was 21.8 months (ranging from 12.8 to 31.7) (Fig. 1d), which were superior to those achieved with chemotherapy in advanced NSCLC patients.

**Fig. 1:**
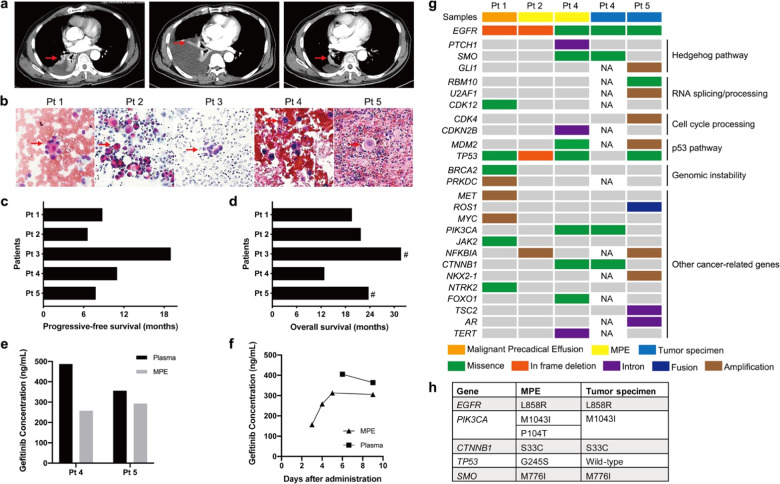
**a** Representative chest computed tomography (CT) from patient 3. Left, baseline CT scan at initial diagnosis. Middle, MPE progression on day 30 after first-line gefitinib administration. Right, CT scan three months after local intervention and continuous gefitinib treatment. Red arrows indicate primary lung lesions. **b** Hematoxylin & eosin (HE) staining of the pleural effusion smear. Samples from patients 2 and 5 were obtained at initial diagnosis, and the other three samples were obtained at early MPE progression. The red arrow indicates malignant cells. Progressive-free survival (PFS) (**c**) and overall survival (OS) (**d**) after first-line gefitinib treatment. #, patients alive at the last follow-up. **e** Gefitinib concentrations in paired MPE and blood samples from patients 4 and 5. Samples were obtained when the MPE progressed after gefitinib treatment (day 41 for patient 4 and day 39 for patient 5). **f** Gefitinib concentration versus time plots of one gefitinib-sensitive patient. **g** Genetic alteration analysis of next-generation sequencing (NGS) results. NA, not available. **h** Comparison of genetic mutations between paired MPE and tumor tissues of patient 4

A previous study reported that MPE progression in *EGFR*-mutant NSCLC patients with target lesion shrinkage followed by gefitinib treatment was ascribed to a possible pleural space barrier that affected drug transport.^1^ In this study, we first detected the gefitinib concentrations in pleural effusion and paired peripheral blood samples by LC-MS/MS. As shown in Fig. 1e, gefitinib was confirmed to penetrate and accumulate in pleural effusion. The mean drug concentrations in MPE were 257.66 and 293.0 ng/mL in patients 4 and 5, respectively. A previous study reported that there was no significant difference in the response rates of gefitinib among different drug concentrations (ranging from 140 to 928 ng/mL) in *EGFR*-mutated NSCLC patients.^2^ In this study, pleural effusion and blood samples from one *EGFR*-mutant LUAD patient who was diagnosed with MPE and showed good response to gefitinib were collected as gefitinib-sensitive controls. In this patient, the gefitinib concentration in the MPE increased gradually after the first administration (day 1), peaked on day 5, and remained stable afterward (Fig. 1f). It was 305.6 ng/mL on day 8, which was similar to that in patients 4 and 5. Therefore, the differences in gefitinib concentrations between MPE and blood should not be a reason for early MPE progression.

To further investigate whether MPE progression could be attributed to the presence of drug-resistant mutations, next-generation sequencing (NGS) was conducted with a commercially available 520 cancer-related gene kit (OncoScreen Plus^TM^, Burning Rock Biotech Ltd, China). DNA was extracted from formalin-fixed paraffin-embedded (FFPE) samples from 4 patients using the QIAamp DNA FFPE Tissue Kit (Qiagen, Hilden, Germany). Sequencing was performed on a NextSeq 500 sequencer (Illumina, Inc., US). Cooccurring genetic aberrations and a median tumor mutation burden (TMB) of 6.0 mutations/Mb (ranging from 2.4 to 12.7 mutations/Mb) were observed (Tables S3, S4), and these alterations involved genes affecting key signaling pathways (such as Hedgehog (*PTCH1, SMO*, and *GLI1*) and P53 (*MDM2*)), genomic instability (*BRCA2* and *PRKDC*), cell cycle processes (*CDK4*), and RNA splicing and processing (*RBM10, U2AF1*, and *CDK12*) (Fig. 1g). Notably, the analysis revealed the coexistence of activating *EGFR* mutations with other driver gene alterations, including alteration of *PIK3CA* and *CTNNB1*, *MET* amplification and *ROS1* fusion. In the current study, we were unable to perform the 520 cancer-related gene test in paired tumor tissues because of insufficient samples. However, paired tumor specimens from patient 4 at the initial diagnosis were analyzed using an NGS panel consisting of 56 lung cancer-related genes (Burning Rock Biotech Ltd, China). The genetic information of paired MPE and tumor specimens showed that concomitant driver mutations occurred in both primary lesions and hydrothorax malignant cells, but the cancer cells in the MPE developed further genetic mutations (Fig. 1g, h). Interestingly, concomitant driver mutations have been previously reported to be an intrinsic resistance mechanism to EGFR-TKIs that can free tumor cells from EGFR signaling dependence by activating multiple bypass pathways. In this study, comutations led to the early progression of only the MPE but not primary lesions after gefitinib treatment, which was most likely due to the discordance of *EGFR* mutation status between paired MPE and tumor tissues.^3^ However, what cannot be explained is that after local treatment, continuous EGFR-TKI administration still showed long-term control of the MPE. One reasonable explanation is that cancer cells continue to be highly addicted to the EGFR pathway despite the existence of other driver mutations. There is evidence that a high response rate (80%) was achieved by EGFR-TKI treatment in NSCLC patients with coexisting EGFR mutations and ALK rearrangements,^4^ which strongly supports *EGFR* mutation as a predominant activation signal in lung cancer.

In addition, we previously reported that the MPE microenvironment could induce lung cancer cells to undergo epithelial-to-mesenchymal transition (EMT) and acquire stem cell properties,^5^ which may further lead to gefitinib resistance. Furthermore, several studies have reported that *PIK3CA* mutation and Hh signaling activation promote the EMT process in NSCLC. In this study, pleural effusion drainage and intrapleural chemotherapy eliminated the impact of the MPE microenvironment on tumor cells, resulting in MPE control and a durable response to gefitinib.

MPE progression after early treatment with EGFR-TKIs represents an uncommon but clinically significant condition. However, considering the small number of clinical cases, it was difficult for us to recruit more patients for a prospective investigation. Further studies on signal transduction are challenging but required. Nevertheless, this study provides important information for clinical decision-making and shows that MPE progression after early treatment with EGFR-TKIs does not represent disease progression, and patients may benefit from continuous TKI treatment after necessary local treatment.

## Supplementary information

Supplementary Materials
