# Supplementary material for: Progression of malignant pleural effusion during the early stage of gefitinib treatment in advanced EGFR-mutant lung adenocarcinoma involving complex driver gene mutations
Source: Signal Transduct Target Ther. 2020 May 27;5:63. doi: 10.1038/s41392-020-0161-7 (PMC7250885; doi:10.1038/s41392-020-0161-7)
Supplement: Supplementary file 1 — Supplementary Materials [file 41392_2020_161_MOESM1_ESM.docx]

Supplementary Materials for

Progression of Malignant Pleural Effusion during Early Stage of Gefitinib Treatment Involving Complex Driver Gene Mutations in Advanced *EGFR*-Mutant Lung Adenocarcinoma

Ning Liu^1^, Min Yu^1^, Tao Yin^2^, Yong Jiang^3^, Xuelian Liao^1^, Jie Tang^4^, Yanying Li^1^, Diyuan Qin^1^, Dan Li^5*^, Yongsheng Wang^1,6*^

Correspondence to: [wangys@scu.edu.cn](mailto:wangys@scu.edu.cn)

**This PDF file includes:**

Tables S1 to S4

Table S1.

Clinical characteristics of lung adenocarcinoma (LUAD) patients with malignant pleural effusion (MPE).

| Patient | Gender | Age(yr) | Smoking status | Stage | Baseline *EGFR* mutation^a^ | Days to MPE progression |
| --- | --- | --- | --- | --- | --- | --- |
| 1 | Male | 56 | Smoker | T4N3M1 | Exon 19 deletion | 5 |
| 2 | Male | 64 | Smoker | T4N2M1 | Exon 19 deletion | 7 |
| 3 | Male | 68 | Nonsomoker | T3NxM1 | Exon 19 deletion | 30 |
| 4 | Male | 67 | Smoker | T4N3M1 | Exon 21 L858R | 20 |
| 5 | Female | 66 | Nonsomoker | T2N0M1 | Exon 21 L858R | 33 |

NOTE: Days to MPE progression was calculated from the first day of gefitinib administration to pleural effusion increase.

^a^Baseline *EGFR* mutations detection was performed on tumor specimens for patients 1, 4 and 5. MPE- and blood-based detection of baseline *EGFR* mutations were used for patients 2 and 3, respectively.

Table S2.

Local treatment and clinical outcomes of lung adenocarcinoma (LUAD)-related MPE.

| Patient | Pt 1 | Pt 2 | Pt 3 | Pt 4 | Pt 5 |
| --- | --- | --- | --- | --- | --- |
| Intrapleural chemotherapy | DDP | DDP | DDP | None | DDP |
| Response evaluation | CR | PR | PR | PR | PR |
| Time to recurrence (months) | - | 19 | - | 11 | - |

DDP, cisplatin; CR, complete response; PR, partial response; -, No MPE recurrence.

**Table S3.**

List of tumor samples available and genetic mutations.

| Patient | Sample | Gene | Variant type | Description | Mutant allele fraction (%) | Copy number |
| --- | --- | --- | --- | --- | --- | --- |
| Pt 1 | Malignant precardical effusion cell block | *BRCA2* | Missense | p.T2403S | 21.74 | / |
|  |  | *CDK12* | Missense | p.H1141Y | 18.31 | / |
|  |  | *TP53* | Missense | p.R273C | 24.69 | / |
|  |  | *PRKAR1A* | Fusion | PRKAR1A-LOC101927876 | 1.56 | / |
|  |  | *LRP1B* | Missense | p.C4018F | 18.15 | / |
|  |  | *TP63* | Missense | p.P476L | 15.50 | / |
|  |  | *EGFR* | Deletion | p.L747_A750del | 36.76 | / |
|  |  | *JAK2* | Missense | p.R300S | 44.32 | / |
|  |  | *PTPRD* | Missense | p.T977S | 12.17 | / |
|  |  | *NTRK2* | Missense | p.W469C | 37.40 | / |
|  |  | *RBM10* | Missense | p.P338Q | 33.68 | / |
|  |  | *MET* | Amplification | / | / | 4.51 |
|  |  | *PRKDC* | Amplification | / | / | 3.87 |
|  |  | *MYC* | Amplification | / | / | 3.44 |
| Pt 2 | MPE cell block | *NFKBIA* | Amplification | / | / | 4.37 |
|  |  | *TP53* | Deletion | p.C124_T125delinsS | 5.22 | / |
|  |  | *EGFR* | Deletion | p.L747_T751del | 17.47 | / |
| Pt 4 | MPE cell block | *ARID2* | Stop_gained | p.Q1096* | 3.81 | / |
|  |  | *MDM2* | Missense | p.E151Q | 8.11 | / |
|  |  | *FOXO1* | Missense | p.S345F | 3.86 | / |
|  |  | *TP53* | Missense | p.G245S | 14.94 | / |
|  |  | *BRIP1* | Missense | p.S986F | 8.00 | / |
|  |  | *CTNNB1* | Missense | p.S33C | 6.87 | / |
|  |  | *PIK3CA* | Missense | p.P104T | 8.61 | / |
|  |  | *PIK3CA* | Missense | p.M1043I | 6.86 | / |
|  |  | *TERT* | Intron | c.2970+12G>A | 7.10 | / |
|  |  | *NPM1* | Intron | c.847-18_847-16del | 9.09 | / |
|  |  | *EGFR* | Missense | p.L858R | 17.73 | / |
|  |  | *SMO* | Missense | p.M776I | 7.39 | / |
|  |  | *CDKN2B* | Intron | c.157-11C>T | 5.84 | / |
|  |  | *PTCH1* | Intron | c.1728+17G>A | 7.17 | / |
| Pt 5 | Tumor specimen | *EGFR* | Missense | p.L858R | 36.28 | / |
|  |  | *TSC2* | Intron | c.1443+14C>T | 5.96 | / |
|  |  | *TP53* | Missense | p.R248W | 1.04 | / |
|  |  | *HIST1H1C* | Missense | p.R54H | 3.46 | / |
|  |  | *ROS1* | Fusion | *GOPC*-*ROS1* | 6.71 | / |
|  |  | *RBM10* | Missense | p.F412C | 30.84 | / |
|  |  | *AR* | Intron | c.2174-19T>G | 12.84 | / |
|  |  | *GLI1* | Amplification | / | / | 4.5 |
|  |  | *CDK4* | Amplification | / | / | 5.3 |
|  |  | *MDM2* | Amplification | / | / | 13.04 |
|  |  | *NFKBIA* | Amplification | / | / | 5.08 |
|  |  | *NKX2-1* | Amplification | / | / | 4.68 |
|  |  | *TMPRSS2* | Amplification | / | / | 4.45 |
|  |  | *U2AF1* | Amplification | / | / | 3.44 |
| /, not applicable. | | | | | | |

**Table S4.**

List of tumor mutation burden (TMB).

| Patient | Pt 1 | Pt 2 | Pt 4 | Pt 5 |
| --- | --- | --- | --- | --- |
| TMB (mutations/Mb) | 7.1 | 2.4 | 12.7 | 4.8 |
